# Mental Health Outcomes Among Civil Servants Aiding in Coronavirus Disease 2019 Control

**DOI:** 10.3389/fpubh.2021.601791

**Published:** 2021-04-29

**Authors:** Qingqing Hu, Xiaochu Hu, Beibei Zheng, Lanjuan Li

**Affiliations:** ^1^State Key Laboratory for Diagnosis and Treatment of Infectious Diseases, National Clinical Research Center for Infectious Diseases, Collaborative Innovation Center for Diagnosis and Treatment of Infectious Diseases, College of Medicine, The First Affiliated Hospital, Zhejiang University School of Medicine, Hangzhou, China; ^2^Department of Oncology, The Affiliated Huai'an No. 1 Hospital of Nanjing Medical University, Nanjing Medical University, Huai'an, China; ^3^Department of Oncology, The Affiliated Nanjing Chest Hospital of Nanjing Medical University, Nanjing Medical University, Nanjing, China

**Keywords:** depression, anxiety, psychiatry, civil servant, cross-sectional study, COVID-19

## Abstract

**Objective:** To assess the psychiatric status of Chinese civil servants aiding in coronavirus disease 2019 (COVID-19) control.

**Background:** During the COVID-19 pandemic, Chinese civil servants have faced high workloads that may contribute to mental disorders. We assessed the prevalence of both depression and anxiety symptoms among civil servants in Jiangsu and surrounding provinces using the Chinese versions of the 9-item Patient Health Questionnaire (PHQ-9) and the 7-item Generalized Anxiety Disorder (GAD-7) scale.

**Methods:** The PHQ-9 and GAD-7 were used to assess the severity of symptoms of depression and anxiety, respectively. Multivariable logistic regression analysis was performed to identify factors associated with mental health outcomes.

**Results:** In total, 867 Chinese civil servants aiding in COVID-19 control were included in our study. Overall, 37.25 and 38.06% of all respondents reported having symptoms of depression and anxiety, respectively. Respondents who were younger and more educated and those who had fewer years of work experience had higher scores for both depression and anxiety. Multivariable logistic regression analysis showed that being a woman, being younger, having more education and having fewer years of work experience were associated with a higher risk of symptoms of depression and anxiety. However, whether they had experience combating infectious diseases or worked in frontline, there was no significant difference between respondents with and without experience, as well as between frontline and non-frontline workers, in both symptoms of depression and anxiety.

**Conclusions:** The civil servants aiding in COVID-19 control reported suffering from varying degrees of mental disorders. Therefore, more attention should be devoted to the psychological distress of these civil servants.

## Introduction

In December 2019, an unknown coronavirus was reported in the Chinese city of Wuhan that causes a novel pneumonia, which was named coronavirus disease 2019 (COVID-19), and more than 80,000 people have been diagnosed with this disease (1). This novel coronavirus pneumonia is so severe that China considers it a level B infectious disease and has adopted strict measures as if it were a level A infectious disease to prevent and control transmission (2). Facing this critical situation, the government, including municipal, county and township governments, has mobilized a large number of officials who belong to different departments, such as public health, transportation, and community associations, to deploy and carry out numerous tasks for controlling the spread of COVID-19. Civil servants on the frontline who were directly involved in controlling the spread of COVID-19 were at risk of developing psychological problems and other mental health symptoms. The rapidly rising number of confirmed and suspected cases, overwhelming workload, shortages of personal protective equipment, and uncooperative citizens can cause psychological stress to these workers. These sudden changes can disrupt a person's daily routine, leading to negative emotions, and psychiatric disorders.

Occupational stress has been proven to cause diverse mental disorders, such as depression and anxiety (2, 3). Depression and anxiety were the third and ninth leading causes of disability in the world, respectively, and may cause a serious decline in work efficiency (4, 5). It has been reported that working long hours (6), lack of public support, high job strain level, work insecurity and an uncomfortable physical environment are risk factors for the development of symptoms of depression and anxiety in civil servants, especially in women (6–8). In addition, many civil servants experience emotional disturbances from mental disorders, including depression and anxiety, during and even after epidemics (9). Therefore, it is essential that psychological assessment and intervention for civil servants occur during large-scale epidemics, leading to the early action of psychological intervention and dramatically improved pandemic control and even rapid social recovery (10).

However, there is currently less information about the psychological impact on civil servants during the 2019 coronavirus outbreak and relatively few studies of psychological status and associated factors among civil servants, especially frontline workers, compared to other COVID-19 response workers. Thus, this study aimed to evaluate the psychological state of frontline civil servants by quantifying the magnitude of depression and anxiety symptoms and analyzing potential risk factors related to these symptoms during COVID-19. This research may provide an assessment of the mental health status of Chinese civil servants, which may provide fundamental evidence to direct the promotion of mental well-being among civil servants.

## Method

### Study Design

The study was conducted during the post-pandemic period of COVID-19, from April 9, 2020, to April 11, 2020, and was based on an online questionnaire survey. Through convenience sampling, a total of 867 frontline civil servants mainly in Jiangsu and surrounding provinces were enrolled in this survey. The inclusion criteria were as follows: adults who were proficient in operating mobile phones to fill in the questionnaire and who were informed and agreed to participate in this study. The exclusion criteria were as follows: individuals who were under the age of 18 and anyone who was not willing to participate or not familiar with cell phones to fill in the questionnaire. Approval (Number: 2020IIT 246) from the clinical research ethics committee of the First Affliated Hospital of Zhejiang University was received before the initiation of this study. An anonymous questionnaire was structured by “WENJUANXING” (https://www.wjx.cn/) and made accessible to individuals via WeChat to reduce face-to-face contact and communication. In addition, we added an informed consent section on the first page of the questionnaire. If the participant disagreed with this section, the questionnaire did not proceed to the next page. Participants could terminate taking the survey at any time, and the information was kept confidential.

### Demographic Data and Comparison of the Diagnosis of Measurements

#### Depression (PHQ-9) and Anxiety (GAD-7)

Demographic data included sex, age, job role, years of work experience, educational background, marriage status, experience, and training for COVID-19. We focused on symptoms of depression and anxiety for all participants. Accordingly, the 9-item Patient Health Questionnaire (PHQ-9; range, 0–27) (11) and the 10-item Generalized Anxiety Disorder (GAD-7) scale (range, 0–21) (12) were used to assess the severity of symptoms of depression and anxiety, respectively. The total scores of these measurement tools were interpreted as follows: PHQ-9, normal (0–4), mild (5–9), moderate (10–14), and severe (15–21) depression; GAD-7, normal (0–4), mild (5–9), moderate (10–14), and severe (15–21) anxiety.

### Statistical Analysis

In this survey, the median and interquartile range (IQR: 25–75%) were used to describe the continuous and non-normally distributed data, whereas the Mann–Whitney *U*-test or Kruskal–Wallis H-test was utilized to measure group differences. Descriptive statistics involved frequencies (%) for categorical variables, and the chi-square test was used to assess group differences. Analyses were performed using SPSS version 20.0 (IBM Corp.), and the data were considered statistically significant when *P* < 0.05.

### Quality Control

The Cronbach's alpha values of PHQ-9 and GAD-7 were 0.892 and 0.847, respectively. The cumulative variance contribution rate of PHQ-9 and GAD-7 reached 64.852 and 65.694%, respectively, which also indicates positive validity. A Spearman correlation analysis was used to determine that the correlation coefficient between the GAD-7 anxiety score and the PHQ-9 depression score was 0.374 (*P* < 0.01), indicating the correlation between depression and anxiety among anti-epidemic public servants after the peak of epidemic.

## Results

### Demographic Characteristics and Comparison of the Diagnosis of Measurements

The comparison between groups of demographic information, exposure history, and psychiatric symptoms, including depressive mood and anxiety, is shown in Table 1. In the study, a total of 867 civil servants were recruited in the study, and 867 individuals (66.7%) completed the survey. Among all the respondents, 470 were women (54.2%), and 397 were men (45.8%). The number of people who were under age 24 was 131 (15.1%), 25 to 29 years, 108 (12.4%), 30 to 34 years, 127 (14.7%), 35 to 39 years, 167 (19.3%), and older than 40 years, 334 (38.5%). The number of unmarried people was 199 (22.95%); married 649 (74.86%); and widowed or divorced 19 (2.19%); the education of the individuals was junior high school, 371 (42.79%), junior college, 274 (31.6%), undergraduate, 211 (24.34%), and graduate, 11 (1.27%). Of the participants, 765 people (88.2%) worked on the frontline and had close contact with crowds, and 261 (20.8%) were administrative personnel. In terms of length of service, there were 246 employees who had worked for <5 years (28.37%), 205 employees who had worked for 5 to 10 years (23.64%), 231 employees who hadworked for 10 to 20 years, (26.64%), and more than 185 employees who had worked for 20 years (21.34%). Among them, 78 (9%) had prior work experience with two or more epidemics, 85 (9.8%) had experience with one epidemic, and 704 (81.2%) had no experience. There were 538 people (62.05%) who had participated in COVID-19-related training and 329 (37.95%) who had not participated in COVID-19-related training. Nearly all participants [799 (92.1%)] lived in Jiangsu Province, and 68 (7.9%) lived in the other 14 provinces. Quite a few respondents showed symptoms of depression [323 (37.25%)] and anxiety [330 (38.06%)]. Age, educational background, and years of work pointed to a significant statistical difference between symptoms of depression, whereas age and years of work experience were the factors related to anxiety. Furthermore, the results showed that there was no difference between frontline and non-frontline workers, experienced and inexperienced individuals, or trained and untrained civil servants.

**Table 1:** Sociodemographic and psychological characteristics of the study participants.

		**No. (%)**						
		**Depression**		**Anxiety**		**Total**	***P*****-value**	
		**Yes**	**No**	**Yes**	**No**		**PHQ9**	**GAD7**
Sex	Men	258 (47.43)	139 (43.03)	244 (45.44)	153 (46.36)	397	0.209	0.79
	Women	286 (2.57)	184 (56.97)	293 (54.56)	177 (53.64)	470		
Age	≤24	63 (19.50)	68 (12.50)	61 (18.48)	70 (13.04)	131	0.03	0.02
	25–29	43 (13.31)	65 (11.95)	50 (15.15)	58 (10.80)	108		
	30–34	42 (13.00)	85 (15.63)	46 (13.94)	81 (15.08)	127		
	35–39	65 (20.12)	102 (18.75)	51 (15.45)	116 (21.60)	167		
	≥40	110 (34.06)	224 (41.18)	122 (36.97)	212 (39.48)	334		
Marriage	Divorced	5 (1.55)	14 (2.57)	8 (2.42)	11 (2.05)	19	2.965	0.227
	Single	83 (25.70)	116 (21.32)	92 (27.88)	107 (19.93)	199		
	Married	235 (72.76)	414 (76.10)	230 (69.70)	419 (78.03)	649		
Educational background	Undergraduate	98 (30.34)	113 (20.77)	93 (28.18)	118 (21.97)	211	<0.01	0.21
	Junior college	115 (35.60)	159 (29.23)	102 (30.91)	172 (32.03)	274		
	High school	105 (32.51)	266 (48.90)	131 (39.70)	240 (44.69)	371		
	Graduate	5 (1.55)	6 (1.10)	4 (1.21)	7 (1.30)	11		
Occupation	Second line	35 (10.84)	67 (12.32)	33 (10.00)	69 (12.85)	102	0.513	0.206
	Frontline	288 (89.16)	477 (87.68)	297 (90.00)	468 (87.15)	765		
Working years	10–20 y	93 (25.91)	138 (27.17)	71 (21.52)	160 (29.80)	231	<0.01	0.04
	5–10 y	53 (14.76)	152 (29.92)	79 (23.94)	126 (23.46)	205		
	≤5 y	141 (39.28)	105 (20.67)	106 (32.12)	140 (26.07)	246		
	≥20 y	72 (20.06)	113 (22.24)	74 (22.42)	111 (20.67)	185		
Experienced	1	36 (11.15)	49 (9.01)	35 (10.61)	50 (9.31)	85	0.58	0.76
	≥2	28 (8.67)	50 (9.19)	31 (9.39)	47 (8.75)	78		
	None	259 (80.19)	445 (81.80)	264 (80.00)	440 (81.94)	704		
Trained or not	Yes	200 (61.92)	338 (62.13)	202 (61.21)	336 (62.57)	538	0.95	0.68
	No	123 (38.08)	206 (37.87)	128 (38.79)	201 (37.43)	329		

### Comparison of the Severity of Measurements and Associated Factors

The PHQ-9 and GAD-7 scores showed a significant difference between different subgroups (Table 2). Younger individuals, well-educated workers, and those who were not middle-ranked for years of work experience reported more severe symptoms of depression, whereas younger individuals, divorced workers, and those who were not middle-ranked for years of work experience showed more severe symptoms of anxiety [e.g., severe depression among younger (25–29) vs. older individuals (≥40): 8 (7.41%) vs. 10 (3.00%); *P* = 0.019; severe anxiety among younger (25–29) vs. older individuals (≥40): 3 (2.78%) vs. 6 (1.80%); *P* = 0.02; severe depression among civil servants who were undergraduate vs. high school educated: 6 (2.85%) vs. 4 (1.08%); *P* = 0.000; severe depression among civil servants who worked ≥20 years vs. 5–10 years: 8 (4.32%) vs. 6 (2.92%); *P* = 0.002; severe anxiety among civil servants who worked ≥20 years vs. 5–10 years: 4 (2.16%) vs. 2 (0.98%); *P* = 0.029; and severe anxiety of civil servants who were divorced vs. married: 1 (5.26%) vs. 6 (0.92%); *P* = 0.018].

**Table 2:** Severity categories of depression and anxiety measurements in the total cohort and subgroups.

		**Sex**			**Age**					
		**No. (%)**			**No. (%)**					
**Severity category**	**Total No. (%)**	**Men**	**Women**	***P*-value**	**≤24**	**25–29**	**30–34**	**35–39**	**≥40**	***P*-value**
**PHQ-9, depression symptoms**										
Normal	544 (62.75)	258 (64.99)	286 (60.85)	0.241	68 (51.91)	65 (60.19)	85 (66.93)	102 (61.08)	224 (67.07)	0.019
Mild	232 (26.76)	100 (25.19)	132 (28.09)		42 (32.06)	28 (25.93)	30 (23.62)	50 (29.94)	82 (24.55)	
Moderate	66 (7.61)	24 (6.05)	42 (8.94)		17 (12.98)	7 (6.48)	11 (8.66)	13 (7.78)	18 (5.39)	
Severe	25 (2.88)	15 (3.78)	10 (2.13)		4 (3.05)	8 (7.41)	1 (0.79)	2 (1.20)	10 (3.00)	
**GAD-7, anxiety**										
Normal	537 (61.94)	244 (61.46)	293 (62.34)	0.676	70 (53.44)	58 (53.70)	81 (63.78)	116 (69.46)	212 (63.47)	0.02
Mild	295 (34.03)	134 (33.75)	161 (34.26)		54 (41.22)	44 (40.74)	41 (32.28)	45 (26.95)	111 (33.23)	
Moderate	23 (2.65)	11 (2.77)	12 (2.55)		5 (3.82)	3 (2.78)	5 (3.94)	5 (2.99)	5 (1.50)	
Severe	12 (1.38)	8 (2.02)	4 (0.86)		2 (1.52)	3 (2.78)	0 (0.00)	1 (0.60)	6 (1.80)	
		**Educational background**						**Working position**		
		**No. (%)**						**No. (%)**		
**Severity category**	**Total No. (%)**	**Bachelor degree**	**Junior college**	**Senior high school**	**Master degree**	***P*****-value**		**Second-line**	**Frontline**	***P*****-value**
**PHQ-9, depression symptoms**										
Normal	544 (62.75)	113 (53.55)	159 (58.03)	266 (71.70)	6 (54.55)	0.000		67 (65.69)	477 (62.35)	0.326
Mild	232 (26.76)	64 (30.33)	78 (28.47)	86 (23.18)	4 (36.36)			29 (28.43)	203 (26.54)	
Moderate	66 (7.61)	28 (13.27)	22 (8.03)	15 (4.04)	1 (9.09)			6 (5.88)	60 (7.84)	
Severe	25 (2.88)	6 (2.85)	15 (5.47)	4 (1.08)	0 (0.00)			0 (0.00)	25 (3.27)	
**GAD-7, anxiety**										
Normal	537 (61.94)	118 (55.92)	172 (62.77)	240 (64.69)	7 (63.64)	0.156		69 (67.65)	468 (61.18)	0.125
Mild	295 (34.03)	81 (38.39)	89 (32.48)	121 (32.61)	4 (36.36)			33 (32.35)	262 (34.25)	
Moderate	23 (2.65)	7 (3.32)	8 (2.92)	8 (2.16)	0 (0.00)			0 (0.00)	23 (3.01)	
Severe	12 (1.38)	5 (2.37)	5 (1.82)	2 (0.54)	0 (0.00)			0 (0.00)	12 (1.48)	
		**Length of service**					**Marriage**			
		**No. (%)**					**No. (%)**			
**Severity category**	**Total No. (%)**	**≤5y**	**5–10 y**	**10–20 y**	**≥20 y**	***P*****-value**	**Divorced**	**Single**	**Married**	***P*****-value**
**PHQ-9, depression symptoms**										
Normal	544 (62.75)	141 (57.32)	152 (74.15)	138 (59.74)	113 (61.08)	0.002	14 (73.68)	116 (58.29)	414 (63.79)	0.195
Mild	232 (26.76)	70 (28.46)	38 (18.54)	71 (30.74)	53 (28.65)		3 (15.79)	56 (28.14)	173 (26.66)	
Moderate	66 (7.61)	27 (10.98)	9 (4.39)	19 (8.23)	11 (5.95)		1 (5.26)	20 (10.05)	45 (6.93)	
Severe	25 (2.88)	8 (3.25)	6 (2.92)	3 (1.30)	8 (4.32)		1 (5.26)	7 (3.52)	17 (2.61)	
**GAD-7, anxiety**										
Normal	537 (61.94)	140 (56.91)	126 (61.46)	160 (69.26)	111 (60.00)	0.029	11 (57.89)	107 (53.77)	419 (64.56)	0.018
Mild	295 (34.03)	94 (38.21)	68 (33.17)	67 (29.00)	66 (35.68)		7 (36.84)	81 (40.70)	207 (31.90)	
Moderate	23 (2.65)	8 (3.25)	9 (4.39)	2 (0.87)	4 (2.16)		0 (0.00)	6 (3.02)	17 (2.62)	
Severe	12 (1.38)	4 (1.63)	2 (0.98)	2 (0.43)	4 (2.16)		1 (5.26)	5 (2.51)	6 (0.92)	
		**Experiences**						**Trained**			
		**No. (%)**						**No. (%)**		
**Severity category**	**Total No. (%)**	**≥2**	**0**	**1**		***P*****-value**		**No**	**Yes**	***P*****-value**
**PHQ-9, depression symptoms**										
Normal	544 (62.75)	50 (64.10)	445 (63.21)	49 (57.65)		0.496		206 (62.61)	338 (62.83)	0.905
Mild	232 (26.76)	20 (25.64)	188 (26.70)	24 (28.24)				91 (27.66)	141 (26.21)	
Moderate	66 (7.61)	5 (6.41)	53 (7.53)	8 (9.41)				26 (7.90)	40 (7.43)	
Severe	25 (2.88)	3 (3.84)	18 (2.56)	4 (4.70)				6 (1.82)	11 (3.53)	
**GAD-7, anxiety**										
Normal	537 (61.94)	47 (60.26)	440 (62.50)	50 (58.82)		0.734		201 (61.09)	336 (62.45)	0.609
Mild	295 (34.03)	29 (37.18)	236 (33.52)	30 (35.29)				112 (34.04)	183 (34.01)	
Moderate	23 (2.65)	1 (1.28)	20 (2.84)	2 (2.35)				13 (3.95)	10 (1.86)	
Severe	12 (1.38)	1 (1.28)	8 (1.13)	3 (3.53)				3 (0.91)	9 (1.67)	

### Comparison of the Scores of Measurements and Associated Factors

The median (IQR) scores on the PHQ-9 for depression and the GAD-7 for anxiety for all participants were 3.0 (1.0–6.0) and 4.0 (2.0–6.0), respectively. Consistently, participants who were younger, well-educated, and those who had fewer years of work experience had higher scores for both depression and anxiety [e.g., median (IQR) PHQ-9 scores among the younger (25–29) vs. the older participants (≥40): 3.0 (1.0–6.0) vs. 2.5 (0.0–6.0); *P* = 0.008; median (IQR) GAD-7 scores among the younger (25–29) vs. the older participants (≥40): 4.0 (3.0–6.0) vs. 4.0 (2.0–6.0); *P* = 0.012; median (IQR) PHQ-9 scores among civil servants who were undergraduates vs. high school educated: 4.0 (2.0–8.0) vs. 2.0 (0.0–5.0); *P* = 0.000; median (IQR) GAD-7 scores among civil servants who were undergraduate vs. high school educated: 4.0 (3.0–6.0) vs. 4.0 (2.0–6.0); *P* = 0.021; median (IQR) PHQ-9 scores among civil servants who worked ≤5 years vs. ≥20 years: 3.0 (1.0–8.0) vs. 3.0 (1.0–6.0); *P* = 0.000; and median (IQR) GAD-7 scores among civil servants who worked ≤5 years vs. ≥20 years: 4.0 (2.0–6.0) vs. 3.0 (1.0–6.0); *P* = 0.041]. However, no significant differences in working position, experience or not and training were noted for the scores of both depression and anxiety (Table 3) [e.g., median (IQR) PHQ-9 scores among experienced (≥2) vs. inexperienced (0): 3.0 (1.0–5.25) vs. 3.0 (1.0–6.0); *P* = 0.857; median (IQR) GAD-7 scores among experienced (≥2) vs. inexperienced (0): 4.0 (2.0–6.0) vs. 4.0 (2.0–6.0); *P* = 0.961; median (IQR) PHQ-9 scores among trained vs. untrained: 3.0 (0.0–6.0) vs. 3.0 (1.0–6.0); *P* = 0.338; and median (IQR) GAD-7 scores among trained vs. untrained: 4.0 (2.0–6.0) vs. 4.0 (2.0–6.0); *P* = 0.478).

**Table 3:** Scores of depression and anxiety measurements in the total cohort and subgroups.

		**Sex**			**Age**					
		**Median (IQR)**			**Median (IQR)**					
**Scale**	**Total score median (IQR)**	**Men**	**Women**	***P*-value**	**≤24**	**25–29**	**30–34**	**35–39**	**≥40**	***P*-value**
PHQ9, depression symptoms	3.0 (1.0–6.0)	3.0 (1.0–6.0)	3.0 (1.0–6.0)	0.590	4.0 (2.0–8.0)	3.0 (1.0–6.0)	2.0 (0.0–6.0)	3.0 (1.0–6.0)	2.5 (0.0–6.0)	0.008
GAD7, anxiety symptoms	4.0 (2.0–6.0)	4.0 (2.0–6.0)	4.0 (2.0–6.0)	0.971	4.0(3.0–6.0)	4.0 (3.0–6.0)	4.0 (2.0–5.0)	3.0 (2.0–5.0)	4.0 (2.0–6.0)	0.012
		**Educational background**					**Working position**			
		**Median(IQR)**					**Median (IQR)**			
**Scale**	**Total score median (IQR)**	**Bachelor degree**	**Junior college**	**Senior high school**	**master degree**	***P*****-value**	**Second-Line**	**Frontline**	***P*****-value**	
PHQ9, depression symptoms	3.0 (1.0–6.0)	4.0 (2.0–8.0)	3.0 (1.0–7.0)	2.0 (0.0–5.0)	3.0 (0.0–8.0)	0.000	3.0 (1.75–6.0)	3.0 (1.0–6.0)	0.433	
GAD7, anxiety symptoms	4.0 (2.0–6.0)	4.0 (3.0–6.0)	3.0 (1.75–6.0)	4.0 (2.0–6.0)	4.0 (2.0–6.0)	0.021	3.0 (2–5.0)	4.0 (2.0–6.0)	0.203	
		**Length of service**					**Marriage**			
		**Median (IQR)**					**Median (IQR)**			
**Scale**	**Total score median (IQR)**	**≤5 y**	**5–10 y**	**10–20 y**	**≥20 y**	***P*****-value**	**Divorced**	**Single**	**Married**	***P*****-value**
PHQ9, depression symptoms	3.0 (1.0–6.0)	3.0 (1.0–8.0)	2.0 (0.0–5.0)	3.0 (1.0–6.0)	3.0 (1.0–6.0)	0.000	2.0 (1.0–5.0)	3.0 (1.0–8.0)	3.0 (1.0–6.0)	0.179
GAD7, anxiety symptoms	4.0 (2.0–6.0)	4.0 (2.0–6.0)	4.0 (2.0–6.0)	3.0 (2.0–5.0)	3.0 (1.0–6.0)	0.041	3.0 (0.0–5.0)	4.0 (3.0–6.0)	4.0 (2.0–6.0)	0.029
		**Experiences**					**Trained**			
		**Median (IQR)**					**Median (IQR)**			
**Scale**	**Total score median(IQR)**	**≥2**	**0**	**1**	***P*****-value**		**No**	**Yes**	***P*****-value**	
PHQ9, depression symptoms	3.0 (1.0–6.0)	3.0 (1.0–5.25)	3.0 (1.0–6.0)	3.0 (1.0–7.0)	0.857		3.0 (1.0–6.0)	3.0 (0.0–6.0)	0.338	
GAD7, anxiety symptoms	4.0 (2.0–6.0)	4.0 (2.0–6.0)	4.0 (2.0–6.0)	3.0 (1.5–6.0)	0.961		4.0 (2.0–6.0)	4.0 (2.0–6.0)	0.478	

### Risk Factors of Mental Health Outcomes

The results of the multivariable logistic regression analysis are presented in Table 4; after controlling for confounders, the variables of being women, being younger, having more education and having fewer years of work experience were associated with severe symptoms of depression and anxiety. In comparison with men, women were associated with more severe symptoms of depression [odds ratio (OR), 1.385; 95% confidence interval (CI), 1.030–1.864; *P* = 0.031] and anxiety (OR, 1.014; 95% CI, 1.008–1.356; *P* = 0.025). The younger participants were associated with a higher risk of feeling distressed (OR, 0.253; 95% CI, 0.117–0.546; *P* = 0.000) and anxious (OR, 0.621; 95% CI, 0.298–0.998; *P* = 0.005) than the older participants. Compared with participants with a high school diploma, those with a college degree appeared to be vulnerable to depression disorders (OR, 2.157; 95% CI, 1.481–3.140; *P* = 0.000). Compared with participants with fewer years of work experience, those with longer years of work experience were associated with severe symptoms of depression (OR, 0.616; 95% CI, 0.383–0.991; *P* = 0.046) and anxiety (OR, 0.755; 95% CI, 0.457–0.947; *P* = 0.032).

**Table 4:** Risk factors associated with depression and anxiety measurements in the total cohort and subgroups.

	**Adjusted OR (95%CI)**		**PHQ9** ***P*****-value**		**GAD7** ***P*****-value**	
**Variable**	**PHQ9**	**GAD7**	**Category**	**Overall**	**Category**	**Overall**
**Sex**						
Men	1 [Reference]	1 [Reference]	NA	0.031	NA	0.025
Women	1.385 (1.030–1.864)	1.014 (1.008–1.356)	0.031		0.025	
**Age**						
≤24	1 [Reference]	1 [Reference]		0.010	NA	0.021
25–29	0.503 (0.277–0.915)	0.740 (0.526–0.980)	0.024		0.035	
30–34	0.294 (0.142–0.611)	0.628 (0.310–0.971)	0.001		0.006	
35–39	0.323 (0.153–0.685)	0.465 (0.222–0.790)	0.003		0.001	
≥40	0.253 (0.117–0.546)	0.621 (0.298–0.998)	0.000		0.005	
**Educational background**						
Senior high school	1 [Reference]	1 [Reference]		0.001	NA	0.021
Junior college	1.753 (1.213–2.534)	0.790 (0.688–0.993)	0.003		0.045	
Undergraduate	2.157 (1.481–3.140)	1.432 (0.990–2.070)	0.000		0.056	
Graduate	2.064 (0.605–7.039)	1.243 (0.769–3.311)	0.247		0.327	
**Working years**						
≤5 y	1 [Reference]	1 [Reference]	NA	0.004	NA	0.002
5–10 y	0.616 (0.383–0.991)	1.041 (0.663–1.633)	0.046		0.063	
10–20 y	0.572 (0.266–1.257)	0.755 (0.457–0.947)	0.353		0.032	
≥20	0.411 (0.244–1.112)	0.773 (0.431–1.179)	0.292		0.314	

## Discussion

Depression is a mood disorder that causes a persistent feeling of sadness and loss of interest (13). The exact cause of depression is unknown. It may be caused by a combination of genetic, biological, environmental, and psychological factors (13). In general, ~1 out of every 6 adults will have depression at some time in their life (14). Symptoms of depression and anxiety often co-occur in certain disorders (15). In fact, it has been estimated that 45 percent of people with one mental health condition meet the criteria for two or more disorders (15). Since the outbreak of COVID-19, increasing studies have shown that this epidemic has a negative impact on the psychology of the general public, and anxiety and depression are the most common mental problems (16–18). In addition, according to the pharmacy benefit management organization Express Scripts, the largest spike was in prescriptions for anti-anxiety drugs, which rose 34.1 percent from mid-February to mid-March 2020, followed by prescriptions for antidepressants that ticked up by 18.6 percent, which also reflect the increase in the incidence of anxiety and depression (19). To date, here have been many reports on the mental stress of medical staff or the public, but there have been few reports on civil servants. Thus, we conducted this study to examine the impact of the COVID-19 pandemic on the prevalence of symptoms of depression and anxiety and the corresponding risk factors among public servants.

This survey enrolled 867 respondents and revealed a high prevalence of mental health symptoms among civil servants during the COVID-19 outbreak in China. Overall, 37.25 and 38.06% of all participants reported symptoms of depression and anxiety, respectively. Respondents who were younger and well-educated and those who had fewer years of work experience had higher scores for both depression and anxiety. Multivariable logistic regression analysis showed that being younger, being a woman, having more education and having fewer years of work experience were associated with severe depression and anxiety. However, the results of the study imply that there is no difference between frontline and non-frontline workers both in depression and anxiety severity among civil servants. This finding suggests that, due to the heavy workload, a large number of civil servants were recruited to the frontline, and the ratio of frontline to second-line personnel may reach 5 to 1 or more, resulting in a reduction in the number of second-line personnel who must perform the tasks that have not been reduced. Thus, both the decreased number of staff and increased tasks caused their workload to rise sharply, leading to the development of the same level of symptoms of both depression and anxiety among frontline and non-frontline workers. These results reached the same conclusion as a previous study (17), indicating that not only frontline nurses but also non-frontline nurses suffered from mental problems. Consistently, during the 2015 MERS outbreak, a study conducted among workers in hospitals reported that not only medical staff who performed MERS-related tasks but also most hospital administrators were reported to be at risk for mental disorders, even after time had elapsed (9).

Judging from the results of whether civil servants had experience or received COVID-19-related training in combating infectious diseases, there was no significant difference between experienced and inexperienced individuals in symptoms of depression or anxiety. This finding may be associated with the fact that the spread rate and impact range of this pandemic are much greater than those of severe acute respiratory syndrome (SARS) in 2003 (20), bringing unimaginable workloads for public officials. Even for responders with experience in administering to public health emergencies, contending with COVID-19 is still a difficult endeavor. Suddenly increased work stress, countless tasks and instructions, fear about the disease, and overwhelming news reports can all cause mental illness in civil servants (21). Furthermore, it has been demonstrated that the presence of both depression and anxiety is common, and working long hours could be a risk factor for the development of depressive and anxiety symptoms, especially in women (6, 22–27). As the epidemic occurred during the Spring Festival, the migration of travelers returning home was extremely fast and widespread, which also increased the difficulty of disease control and increased the workload of civil servants. In addition, although many departments at the state and regional levels have set up COVID-19-related courses, there are few training opportunities on mental health and psychological support. Finally, all of the above factors worked together and eventually led to mental illness.

To address COVID-19, China has taken active and effective strategies to support the prevention and control of the epidemic and has adopted a strict prevention and control system, mobilizing a large number of civil servants to participate in these actions, which have been proven to be highly effective. According to the reports, no new cases in Wuhan were reported for 14 days continuously; thus, with the approval of the Hubei Provincial Government, Wuhan, which is the original place of the outbreak, the road blockade was lifted at 0:00 on April 8, 2020. Compared to many other occupations in China, civil servants are more susceptible to various psychosocial pressures, such as responsibilities, overworking, fierce competition, and complicated interpersonal relationships (28, 29). As a result, they often suffer from psychological problems under high pressure, especially young civil servants, among whom the prevalence of psychiatric symptoms has risen markedly (28–30). Meanwhile, they are also at high risk of psychological distress when exposed to non-cooperation or even violence in the course of managing various issues and interacting with different individuals (31).

How can these workers be helped? Mental health practitioners should carry out psychiatric interventions as early and timely as possible to deal with the outbreak of these severe infectious diseases (32, 33). Prompt and continuous psychological interventions may be beneficial for the mental health of civil servants during high mortality infectious disease outbreaks (21). Civil servants should be provided with more support programs to address the subsequent impact of this severe epidemic. Those most liable to suffer from serious mental illness include women, people of younger age, individuals with more education and individuals with fewer years of job experience. Importantly, these groups require particular attention.

In summary, this study suggests that during the period of the COVID-19 pandemic spread and control, civil servants suffer from depression and anxiety according to varying levels, presenting concerns about the psychological status of civil servants involved in the recent COVID-19 outbreak in China. These findings may improve the understanding of mental health status and various stressors in civil servants and provide a theoretical basis and feasible strategy for future work. Therefore, early recognition of mental disorders and psychological stress, including depression and anxiety in civil servants, can promote the prevention, control, and treatment of COVID-19.

This study is a descriptive and cross-sectional study that is unable to explore the causal relationship between factors. Therefore, it is necessary to conduct longitudinal large-scale intervention studies and recruit more civil servants to further explore the pathogenesis, treatment strategies, and mechanisms of depression and anxiety.

## Data Availability Statement

The original contributions presented in the study are included in the article/Supplementary Material, further inquiries can be directed to the corresponding author/s.

## Ethics Statement

The studies involving human participants were reviewed and approved by the clinical research ethics committee of the First Affiliated Hospital of Zhejiang University. Written informed consent for participation was not required for this study in accordance with the national legislation and the institutional requirements. Written informed consent was not obtained from the individual(s) for the publication of any potentially identifiable images or data included in this article.

## Author Contributions

LL designed the study. QH and BZ supervised data collection and data management. QH and XH analyzed the data. QH prepared the first draft of the manuscript. All authors wrote the paper, had access to the study data, and have reviewed and approved the final manuscript.

## Conflict of Interest

The authors declare that the research was conducted in the absence of any commercial or financial relationships that could be construed as a potential conflict of interest.

## References

[1] Li Q Guan X Wu P Wang X Zhou L Tong Y. Early transmission dynamics in Wuhan, China, of novel coronavirus-infected pneumonia. N Engl J Med. (2020) 382:1199–1207. 10.1056/NEJMoa2001316 31995857 PMC7121484

[2] Stansfeld S Candy B. Psychosocial work environment and mental health–a meta-analytic review. Scand J Work Environ Health. (2006) 32:443–62. 10.5271/sjweh.1050 17173201

[3] Theorell T Hammarström A Aronsson G Träskman Bendz L Grape T Hogstedt C. A systematic review including meta-analysis of work environment and depressive symptoms. BMC Public Health. (2015) 15:738. 10.1186/s12889-015-1954-4 26232123 PMC4522058

[4] Plaisier I Beekman AT de Graaf R Smit JH van Dyck R Penninx BW. Work functioning in persons with depressive and anxiety disorders: the role of specific psychopathological characteristics. J Affect Disord. (2010) 125:198–206. 10.1016/j.jad.2010.01.072 20185180

[5] GBD. 2015 Disease and injury incidence and prevalence collaborators. global, regional, and national incidence, prevalence, and years lived with disability for 310 diseases and injuries, 1990-2015: a systematic analysis for the global burden of disease study 2015. Lancet. (2016) 388:1545–602. 10.1016/S0140-6736(16)31678-6 27733282 PMC5055577

[6] Virtanen M Ferrie JE Singh-Manoux A Shipley MJ Stansfeld SA Marmot MG. Long working hours and symptoms of anxiety and depression: a 5-year follow-up of the whitehall II study. Psychol Med. (2011) 41:2485–94. 10.1017/S0033291711000171 21329557 PMC3095591

[7] Aronsson G Theorell T Grape T Hammarström A Hogstedt C Marteinsdottir I. A systematic review including meta-analysis of work environment and burnout symptoms. BMC Public Health. (2017) 17:264. 10.1186/s12889-017-4153-7 28302088 PMC5356239

[8] Guan S Xiaerfuding X Ning L Lian Y Jiang Y Liu J. Effect of job strain on job burnout, mental fatigue and chronic diseases among civil servants in the xinjiang uygur autonomous region of China. Int J Environ Res Public Health. (2017) 14:872. 10.3390/ijerph14080872 28771199 PMC5580576

[9] Lee SM Kang WS Cho AR Kim T Park JK. Psychological impact of the 2015 MERS outbreak on hospital workers and quarantined hemodialysis patients. Compr Psychiatry. (2018) 87:123–27. 10.1016/j.comppsych.2018.10.003 30343247 PMC7094631

[10] Kayama M Akiyama T Ohashi A Horikoshi N Kido Y Murakata T. Experiences of municipal public health nurses following Japan's earthquake, tsunami, and nuclear disaster. Public Health Nurs. (2014) 31:517–25. 10.1111/phn.12140 25040837

[11] Zhang YL Liang W Chen ZM Zhang HM Zhang JH Weng XQ. Validity and reliability of patient health questionnaire-9 and patient health questionnaire-2 to screen for depression among college students in China. Asia Pac Psychiatry. (2013) 5:268–75. 10.1111/appy.12103 24123859

[12] He XY Li CB Qian J Cui HS Wu WY. Reliability and validity of a generalized anxiety scale in general hospital outpatients. Shanghai Arch Psychiatry. 22:200–3. 10.3969/j.issn.1002-0829.2010.04.002

[13] Belmaker RH Agam G. Major depressive disorder. N Engl J Med. (2008) 358:55–68. 10.1056/NEJMra073096 18172175

[14] Kessler RC Berglund P Demler O Jin R Merikangas KR Walters EE. Lifetime prevalence and age-of-onset distributions of DSM-IV disorders in the national comorbidity survey replication. Arch Gen Psychiatry. (2005) 62:593–602. 10.1001/archpsyc.62.6.593 15939837

[15] Kessler RC Birnbaum HG Shahly V Bromet E Hwang I McLaughlin KA. Age differences in the prevalence and co-morbidity of DSM-IV major depressive episodes: results from the WHO world mental health survey initiative. Depress Anxiety. (2010) 27:351–64. 10.1002/da.20634 20037917 PMC3139270

[16] Lai J Ma S Wang Y Cai Z Hu J Wei N. Factors associated with mental health outcomes among health care workers exposed to coronavirus disease 2019. JAMA Netw Open. (2020) 3:e203976. 10.1001/jamanetworkopen.2020.3976 32202646 PMC7090843

[17] Li Z Ge J Yang M Feng J Qiao M Jiang R. Vicarious traumatization in the general public, members, and non-members of medical teams aiding in COVID-19 control. Brain Behav Immun. (2020) 88:916–9. 10.1016/j.bbi.2020.03.007 32169498 PMC7102670

[18] Liu N Zhang F Wei C Jia Y Shang Z Sun L. Prevalence and predictors of PTSS during COVID-19 outbreak in China hardest-hit areas: gender differences matter. Psychiatry Res. (2020) 287:112921. 10.1016/j.psychres.2020.112921 32240896 PMC7102622

[19] Fox News. Prescriptions for Anti-Anxiety Meds Spike Amid Coronavirus Outbreak, New Report Finds. Nick Givas FoxNews. Available online at: https://www.foxnews.com/health/prescriptions-anti-anxiety-meds-spike-amid-coronavirus

[20] Chen N Zhou M Dong X Qu J Gong F Han Y. Epidemiological and clinical characteristics of 99 cases of 2019 novel coronavirus pneumonia in Wuhan, China: a descriptive study. Lancet. (2020) 395:507–13. 10.1016/S0140-6736(20)30211-7 32007143 PMC7135076

[21] Mukhtar S. Psychological health during the coronavirus disease 2019 pandemic outbreak. Int J Soc Psychiatry. (2020) 66:512–6. 10.1177/0020764020925835 32434402 PMC7405632

[22] Bildt C Michélsen H. Gender differences in the effects from working conditions on mental health: a 4-year follow-up. Int Arch Occup Environ Health. (2002) 75:252–8. 10.1007/s00420-001-0299-8 11981659

[23] Sparks K Cooper C Fried Y Shirom A. The effects of hours of work on health: a meta-analytic review. J Occup Organ Psychol. (2011) 70:391–408. 10.1111/j.2044-8325.1997.tb00656.x

[24] Kleppa E Sanne B Tell GS. Working overtime is associated with anxiety and depression: the hordaland health study. J Occup Environ Med. (2008) 50:658–66. 10.1097/JOM.0b013e3181734330 18545093

[25] Park J Kim Y Chung HK Hisanaga N. Long working hours and subjective fatigue symptoms. Ind Health. (2001) 39:250–4. 10.2486/indhealth.39.250 11500001

[26] Shields M. Long working hours and health. Health Rep. (1999) 11:33–48. 10618741

[27] Spurgeon A Harrington JM Cooper CL. Health and safety problems associated with long working hours: a review of the current position. Occup Environ Med. (1997) 54:367–75. 10.1136/oem.54.6.367 9245942 PMC1128796

[28] Zhu C Chen L Ou L Geng Q Jiang W. Relationships of mental health problems with stress among civil servants in Guangzhou, China. Community Ment Health J. (2014) 50:991–6. 10.1007/s10597-014-9726-7 24696152

[29] Liang Y Cao R. Employment assistance policies of Chinese government play positive roles! the impact of post-earthquake employment assistance policies on the health-related quality of life of Chinese earthquake populations. Soc Indic Res. (2015) 120:835–57. 10.1007/s11205-014-0620-z

[30] Liang Y Wang L Yin X. The factor structure of the 12-item general health questionnaire (GHQ-12) in young Chinese civil servants. Health Qual Life Outcomes. (2016) 14:136. 10.1186/s12955-016-0539-y 27669741 PMC5037881

[31] Lopes CS Moraes CL Junger WL Werneck GL Ponce de Leon AC Faerstein E. Direct and indirect exposure to violence and psychological distress among civil servants in Rio de Janeiro, Brazil: a prospective cohort study. BMC Psychiatry. (2015) 15:109. 10.1186/s12888-015-0487-9 25947364 PMC4426549

[32] Mukhtar S. Mental health and psychosocial aspects of coronavirus outbreak in pakistan: psychological intervention for public mental health crisis. Asian J Psychiatry. (2020) 51:102069. 10.1016/j.ajp.2020.102069 32344331 PMC7161472

[33] Srivatsa S Stewart KA. How should clinicians integrate mental health into epidemic responses? AMA J Ethics. (2020) 22:E10–5. 10.1001/amajethics.2020.10 31958385

