# Supplementary material for: Mental Health Outcomes Among Civil Servants Aiding in Coronavirus Disease 2019 Control
Source: Front Public Health. 2021 Apr 29;9:601791. doi: 10.3389/fpubh.2021.601791 (PMC8118382; doi:10.3389/fpubh.2021.601791)
Supplement: Supplementary file 2 [file Table_2.DOCX]

**A survey on psychological stress of public officials on the front line of COVID-19**

**抗疫一线公职人员心理压力调查表**

In this study, you will get some of your psychological information, which is helpful to understand the psychological status of public servants during the epidemic. If you decide to participate in this study, your personal information will be kept strictly confidential, and we will not disclose it to anyone unless we have your permission. In order to ensure the authenticity of the survey, you must provide your real data which is only for research. If necessary, the ethics committee can be granted access to your data according to regulations. As a participant, you can decide whether to continue participating in this survey.

1. Gender: male female
2. Age ≤24 25-29 30-34 35-39 ≥40
3. Marriage status: Divorced Married Single
4. Educational background：

graduate junior college high school undergraduate

1. Occupation: Second-line Front-line
2. Working years: ≤5y 5-10y 10-20y ≥20y
3. Experienced: 1 ≥2 none
4. Trained or not: yes no

Below we will ask you how you feel or think about certain events. Please just choose the first impression. Please choose one of the 4 options for each question below as your answer, thank you! 下面我们将询问您对某些事件的感受或想法。回答时不要思考，选择第一印象即可。请在下面每个问题的4个选择项中选一项作为您的回答，谢谢您！

Not at all Several days Over half the days Nearly every day

1. Little interest or pleasure in doing things
2. Feeling down, depressed, or hopeless
3. Trouble falling or staying asleep, or sleeping too much
4. Feeling tired or having little energy
5. Poor appetite or overeating
6. Feeling bad about yourself or that you are a failure or have let yourself or your family down
7. Trouble concentrating on things, such as reading the newspaper or watching television
8. Moving or speaking so slowly that other people could have noticed. Or the opposite being so figety or restless that you have been moving around a lot more than usual
9. Thoughts that you would be better off dead, or of hurting yourself
10. Feeling nervous, anxious, or on edge
11. Not being able to stop or control worrying
12. Worrying too much about different things
13. Trouble relaxing
14. Being so restless that it’s hard to sit still
15. Becoming easily annoyed or irritable
16. Feeling afraid as if something awful might happen
